# Supplementary material for: COZOID: contact zone identifier for visual analysis of protein-protein interactions
Source: BMC Bioinformatics. 2018 Apr 6;19:125. doi: 10.1186/s12859-018-2113-6 (PMC5889581; doi:10.1186/s12859-018-2113-6)
Supplement: Supplementary file 2 — Software build. Executable binary file of the software tool. (ZIP 118,784 kb) [file 12859_2018_2113_MOESM2_ESM.zip › cozoid/java/docs/beansbinding-1.2.1-doc.zip!/org/jdesktop/el/impl/MethodExpressionLiteral.html]

MethodExpressionLiteral (Beans Binding)


---


|  |  |  |
| --- | --- | --- |
| **Overview** \| **Package** \| **Class** \| **Use** \| **Tree** \| **Deprecated** \| **Index** \| **Help** | |  |
| **PREV CLASS**   **NEXT CLASS** | **FRAMES**    **NO FRAMES**     **All Classes** |  |
| SUMMARY: NESTED \| FIELD \| CONSTR \| METHOD | DETAIL: FIELD \| CONSTR \| METHOD |  |


---


## org.jdesktop.el.impl Class MethodExpressionLiteral

```
java.lang.Object
  org.jdesktop.el.Expression
      org.jdesktop.el.MethodExpression
          org.jdesktop.el.impl.MethodExpressionLiteral
```

**All Implemented Interfaces:**: java.io.Externalizable, java.io.Serializable

---

``` public class MethodExpressionLiteral extends MethodExpression implements java.io.Externalizable ```

**See Also:**: Serialized Form

---

**Nested Class Summary**

**Nested classes/interfaces inherited from class org.jdesktop.el.Expression**

`Expression.ResolvedProperty, Expression.Result`


**Constructor Summary**

`MethodExpressionLiteral()`

`MethodExpressionLiteral(java.lang.String expr,
java.lang.Class expectedType,
java.lang.Class[] paramTypes)`


| **Method Summary** | |
| --- | --- |
| `boolean` | `equals(java.lang.Object obj)`             Determines whether the specified object is equal to this `Expression`. |
| `java.lang.String` | `getExpressionString()`             Returns the original String used to create this `Expression`, unmodified. |
| `MethodInfo` | `getMethodInfo(ELContext context)`             Evaluates the expression relative to the provided context, and returns information about the actual referenced method. |
| `int` | `hashCode()`             Returns the hash code for this `Expression`. |
| `java.lang.Object` | `invoke(ELContext context, java.lang.Object[] params)`             If a String literal is specified as the expression, returns the String literal coerced to the expected return type of the method signature. |
| `boolean` | `isLiteralText()`             Returns whether this expression was created from only literal text. |
| `void` | `readExternal(java.io.ObjectInput in)` |
| `void` | `writeExternal(java.io.ObjectOutput out)` |

**Methods inherited from class java.lang.Object**

`clone, finalize, getClass, notify, notifyAll, toString, wait, wait, wait`

**Constructor Detail**

### MethodExpressionLiteral

```
public MethodExpressionLiteral()
```

---


### MethodExpressionLiteral

```
public MethodExpressionLiteral(java.lang.String expr,
                               java.lang.Class expectedType,
                               java.lang.Class[] paramTypes)
```


**Method Detail**

### getMethodInfo

```
public MethodInfo getMethodInfo(ELContext context)
                         throws ELException
```

:   **Description copied from class: `MethodExpression`**
:   Evaluates the expression relative to the provided context, and
    returns information about the actual referenced method.

    :   **Specified by:**: `getMethodInfo` in class `MethodExpression`
    :   **Parameters:**: `context` - The context of this evaluation **Returns:**: an instance of `MethodInfo` containing information about the method the expression evaluated to. **Throws:**: `PropertyNotFoundException` - if one of the property resolutions failed because a specified variable or property does not exist or is not readable.: `MethodNotFoundException` - if no suitable method can be found.: `ELException` - if an exception was thrown while performing property or variable resolution. The thrown exception must be included as the cause property of this exception, if available.

---


### invoke

```
public java.lang.Object invoke(ELContext context,
                               java.lang.Object[] params)
                        throws ELException
```

:   **Description copied from class: `MethodExpression`**
:   If a String literal is specified as the expression, returns the
    String literal coerced to the expected return type of the method
    signature. An `ELException` is thrown if
    `expectedReturnType` is void or if the coercion of the String literal
    to the `expectedReturnType` yields an error (see Section "1.16 Type
    Conversion" of the EL specification).
    If not a String literal, evaluates the expression
    relative to the provided context, invokes the method that was
    found using the supplied parameters, and returns the result of
    the method invocation.
    Any parameters passed to this method is ignored if isLiteralText()
    is true.

    :   **Specified by:**: `invoke` in class `MethodExpression`
    :   **Parameters:**: `context` - The context of this evaluation.: `params` - The parameters to pass to the method, or `null` if no parameters. **Returns:**: the result of the method invocation (`null` if the method has a `void` return type). **Throws:**: `PropertyNotFoundException` - if one of the property resolutions failed because a specified variable or property does not exist or is not readable.: `MethodNotFoundException` - if no suitable method can be found.: `ELException` - if a String literal is specified and expectedReturnType of the MethodExpression is void or if the coercion of the String literal to the expectedReturnType yields an error (see Section "1.16 Type Conversion").

---


### getExpressionString

```
public java.lang.String getExpressionString()
```

:   **Description copied from class: `Expression`**
:   Returns the original String used to create this `Expression`,
    unmodified.

    This is used for debugging purposes but also for the purposes
    of comparison (e.g. to ensure the expression in a configuration
    file has not changed).

    This method does not provide sufficient information to
    re-create an expression. Two different expressions can have exactly
    the same expression string but different function mappings.
    Serialization should be used to save and restore the state of an
    `Expression`.

    :   **Specified by:**: `getExpressionString` in class `Expression`
    :   **Returns:**: The original expression String.

---


### equals

```
public boolean equals(java.lang.Object obj)
```

:   **Description copied from class: `Expression`**
:   Determines whether the specified object is equal to this
    `Expression`.

    The result is `true` if and only if the argument is
    not `null`, is an `Expression` object that
    is the of the same type (`ValueExpression` or
    `MethodExpression`), and has an identical parsed
    representation.

    Note that two expressions can be equal if their expression
    Strings are different. For example, `${fn1:foo()}`
    and `${fn2:foo()}` are equal if their corresponding
    `FunctionMapper`s mapped `fn1:foo` and
    `fn2:foo` to the same method.

    :   **Specified by:**: `equals` in class `Expression`
    :   **Parameters:**: `obj` - the `Object` to test for equality. **Returns:**: `true` if `obj` equals this `Expression`; `false` otherwise. **See Also:**: `Hashtable`, `Object.equals(java.lang.Object)`

---


### hashCode

```
public int hashCode()
```

:   **Description copied from class: `Expression`**
:   Returns the hash code for this `Expression`.

    See the note in the `Expression.equals(java.lang.Object)` method on how two expressions
    can be equal if their expression Strings are different. Recall that
    if two objects are equal according to the `equals(Object)`
    method, then calling the `hashCode` method on each of the
    two objects must produce the same integer result. Implementations must
    take special note and implement `hashCode` correctly.

    :   **Specified by:**: `hashCode` in class `Expression`
    :   **Returns:**: The hash code for this `Expression`. **See Also:**: `Expression.equals(java.lang.Object)`, `Hashtable`, `Object.hashCode()`

---


### isLiteralText

```
public boolean isLiteralText()
```

:   **Description copied from class: `Expression`**
:   Returns whether this expression was created from only literal text.

    This method must return `true` if and only if the
    expression string this expression was created from contained no
    unescaped EL delimeters (`${...}` or
    `#{...}`).

    :   **Specified by:**: `isLiteralText` in class `Expression`
    :   **Returns:**: `true` if this expression was created from only literal text; `false` otherwise.

---


### readExternal

```
public void readExternal(java.io.ObjectInput in)
                  throws java.io.IOException,
                         java.lang.ClassNotFoundException
```

:   **Specified by:**: `readExternal` in interface `java.io.Externalizable`
:   **Throws:**: `java.io.IOException`: `java.lang.ClassNotFoundException`

---


### writeExternal

```
public void writeExternal(java.io.ObjectOutput out)
                   throws java.io.IOException
```

:   **Specified by:**: `writeExternal` in interface `java.io.Externalizable`
:   **Throws:**: `java.io.IOException`


---


|  |  |  |
| --- | --- | --- |
| **Overview** \| **Package** \| **Class** \| **Use** \| **Tree** \| **Deprecated** \| **Index** \| **Help** | |  |
| **PREV CLASS**   **NEXT CLASS** | **FRAMES**    **NO FRAMES**     **All Classes** |  |
| SUMMARY: NESTED \| FIELD \| CONSTR \| METHOD | DETAIL: FIELD \| CONSTR \| METHOD |  |


---
